# Biofilm Formation and Immunomodulatory Activity of *Proteus mirabilis* Clinically Isolated Strains

**DOI:** 10.3390/ijms18020414

**Published:** 2017-02-15

**Authors:** Alessandra Fusco, Lorena Coretti, Vittoria Savio, Elisabetta Buommino, Francesca Lembo, Giovanna Donnarumma

**Affiliations:** 1 Department of Experimental Medicine, Section of Microbiology and Clinical Microbiology, University of Campania Luigi Vanvitelli, via Costantinopoli 16, 80100 Naples, Italy; alessandra.fusco@unina2.it (A.F.); vittoriasavio@libero.it (V.S.); 2 Institute for Experimental Endocrinology and Oncology “G.Salvatore”, National Research Council (CNR), via Pansini 5, 80131 Naples, Italy; lorena.coretti@tiscali.it; 3 Department of Pharmacy, University of Naples “Federico II”, via D. Montesano 47, 80131 Naples, Italy; elisabetta.buommino@unina.it (E.B.); frlembo@unina.it (F.L.)

**Keywords:** UTI, *Proteus mirabilis*, biofilm

## Abstract

Urinary tract infections (UTIs) and catheter-associated UTIs (CAUTIs) are the principal hospital-acquired infections. *Proteus mirabilis* is characterized by several virulence factors able to promote adhesion and biofilm formation and ameliorate the colonization of urinary tract and the formation of crystalline biofilms on the abiotic surface of the urinary catheters. Since, to date, the role of *P. mirabilis* in the etiopathogenesis of different types of urinary tract infections is not well established, in this study we sought to characterize two different clinically isolated strains of *P. mirabilis* (PM1 and PM2) with distinctive phenotypes and analyzed various virulence factors possibly implicated in the ability to induce UTIs and CAUTIs. In particular, we analyzed motility, biofilm formation both on abiotic and biotic surfaces of PM1 and PM2 and paralleled these parameters with the ability to induce an inflammatory response in an epithelial cell model. Results showed that PM1 displayed major motility and a capacity to form biofilm and was associated with an anti-inflammatory response of host cells. Conversely, PM2 exhibited lack motility and a had slower organization in biofilm but promoted an increase of proinflammatory cytokine expression in infected epithelial cells. Our study provides data useful to start uncovering the pathologic basis of *P. mirabilis*-associated urinary infections. The evidence of different virulence factors expressed by PM1 and PM2 highlights the possibility to use precise and personalized therapies targeting specific virulence pathways.

## 1. Introduction

Urinary tract infections (UTIs) are infection disorders associated with colonization, growth and dissemination of bacteria within the urinary tract. UTIs affect both sexes, although with a different impact depending on the age group. In fact, while in females there is a greater incidence from childhood until the age of 30, in males the frequency is high in the neonatal age and with aging, especially in the presence of prostate disease [1].

In general, UTIs affect 150 million people each year worldwide, representing the most common infection after respiratory and gastro-intestinal infections [2]. UTIs are divided into uncomplicated and complicated, based on the absence or presence of predisposing factors that alter urinary functionality [3]. In particular, catheter-associated UTIs (CAUTIs) have been associated with increased morbidity and mortality with respect to other forms of complicated and uncomplicated UTIs [1].

Most uropathogenic bacteria belong to the intestinal microbiota which colonizes the perianal area of the patient [4]; alternatively, in CAUTIs, catheter insertion and manipulation facilitate the entry into the bladder of opportunistic bacteria not usually considered urinary tract pathogens [5].

Bacterial adhesins recognize molecules on uroepithelial cells [6] as receptors or new binding sites generated by epithelial mucosa damage after catheter placement [5]. Adhesion to the urinary epithelium or the catheter surface frequently induces a phenotypic switch of the bacterial population, resulting in the production of exopolysaccharides and biofilm maturation which, once formed, protects uropathogenic bacteria from the action of antibiotics and the host immune response [7].

Among the microrganisms most commonly found in urinary infections, *Escherichia coli* is highly represented with a rate of 75%–90% of incidence, but the frequency of infections caused by *Proteus* spp. is reported to be increased especially in the nosocomial context, or in complicated infections [8,9]. Among the four *Proteus* species currently recognized, *Proteus mirabilis* is the most frequently isolated in urinary opportunistic infections and poses particular problems in the care of patients undergoing long-term indwelling bladder catheterization [10].

Several virulence factors may be involved in the pathogenicity of *P. mirabilis* in the catheterized urinary tract. Among all, the well-known ability of *P. mirabilis* to swarm rapidly over surfaces allows the rapid migration of “rafts” of *P. mirabilis* cells over the solid surfaces of catheters [11]. On solid surfaces, *P. mirabilis* swimmer cells differentiate into elongated, hyperflagellated swarmers which exhibit an increased expression of virulence factors responsible for improving its ability to adhere to the urinary epithelium or to the polymers of the catheters. Swarming is regulated by more than 50 genes [12]. This differentiation is due to an overexpression of the *wosA* gene, which in turn increases the expression of the *flhDC* activator, a central component in the regulation of swarmer cell differentiation in *P. mirabilis* [12,13]. This heterodimeric complex (*FlhD2C2*), whose expression increases 10-fold during the initiation of swarmer cell differentiation, activates the promoters for Class 2 genes in the flagellar cascade that encode the flagellar basal body and the sigma factor. The expression of σ^28^ allows RNA polymerase to transcribe the Class 3 genes, which include flagellin, resulting in hyperflagellation of the swarmer cells [12].

Another virulence factor is the production of urease which catalyzes the hydrolysis of urea to carbon dioxide and ammonia [14], elevates urine pH and promotes the sedimentation of calcium crystals (apatite) and magnesium ammonium phosphate ammoprecipitates (struvite) in urine and on catheters [15]. Results of this process are the formation of the characteristic crystalline biofilm on catheters and the accumulation of kidney stones of *P. mirabilis* derived from the incorporation of crystals in the polysaccharidic matrices [16,17,18]. *Proteus* crystalline biofilm favors the persistence of microorganisms in the urinary tract, protects from antibiotics and the immune response, and helps with access to the uro-epithelial surfaces, and the accumulation of ammonia becomes toxic for the uro-epithelial cells and induces direct tissue damage [19,20]. Moreover, the characteristic formation of urinary stones during UTI caused by *P*. *mirabilis* can block the flow of urine through the catheter, bladder or kidneys [16], and a defective urine drainage and reflux of urine to the kidneys can result in serious complications and the development of pyelonephritis, septicemia, and shock [15].

In this scenario, a focus on the molecular basis of the virulence of uropathogens, such as *P. mirabilis*, is essential to provide specific therapies targeting specific key virulence pathways.

To this aim, in this work we described two phenotypically different, clinically isolated strains of *P. mirabilis* with the ability to form biofilm, exploring their motility capabilities and analyzing the mRNA expression of genes involved in swarmer cell differentiation. Moreover, we investigated the ability of the two strains to induce an immune response in human urinary bladder transitional cell carcinoma T24 cells. The results of this study motivate us to further investigate the virulence mechanisms of *P. mirabilis* to define its role in UTIs and CAUTIs.

## 2. Results

### 2.1. Characterization of P. mirabilis Strains

**Figure 1:**
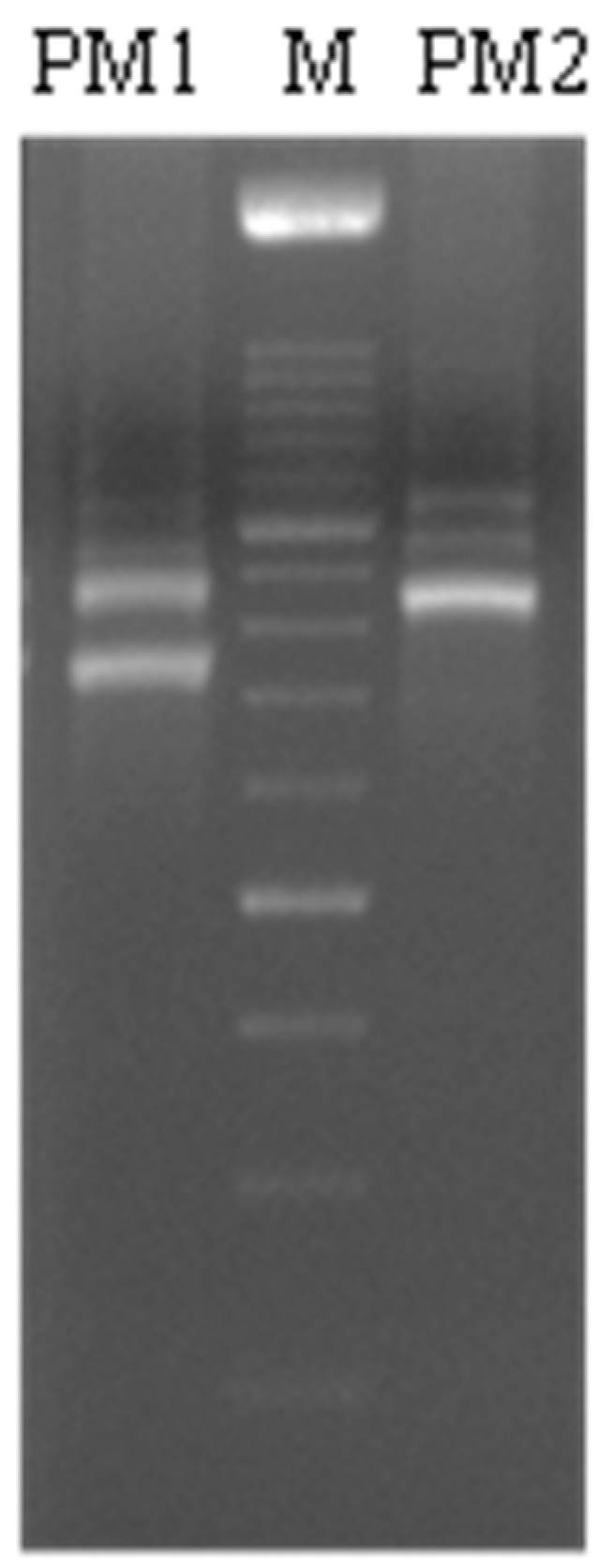
Agarose gel electrophoresis of 16S–23S *Proteus* Internal Transcribed Spacer (ITS) region amplification showing the banding patterns of PM1 and PM2.

**Figure 2:**
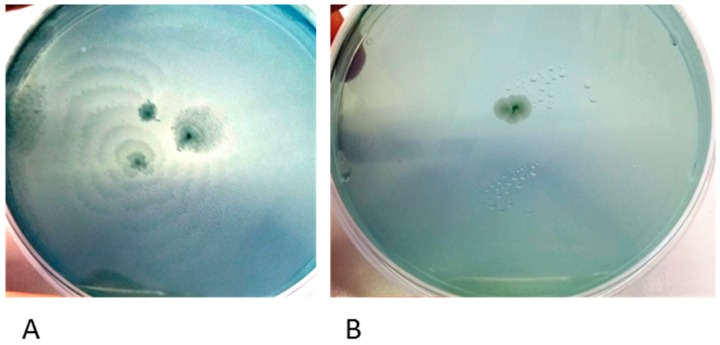
Swarming of PM1 (**A**); and PM2 (**B**) on Cystine Lactose Electrolyte Deficient (CLED) agar plates.

The clinical isolates were biochemically identified through the “API20NE identification system” as *P. mirabilis* and named PM1 and PM2. They were also characterized on a genomic basis and using their colony morphology characteristics. The PCR amplification of the 16S–23S Internal Transcribed Spacer (ITS) sequence showed that the two strains of *P. mirabilis* have different molecular patterns (Figure 1). In addition, on Cystine Lactose Electrolyte Deficient (CLED) agar plates they showed a different phenotype (Figure 2); PM1 exhibited swarming motility, while PM2 did not; so, they were defined “swarmer” and “non-swarmer”, respectively.

### 2.2. Biofilm Formation, Evaluation of Motility and Expression of the wosA Gene

**Figure 3:**
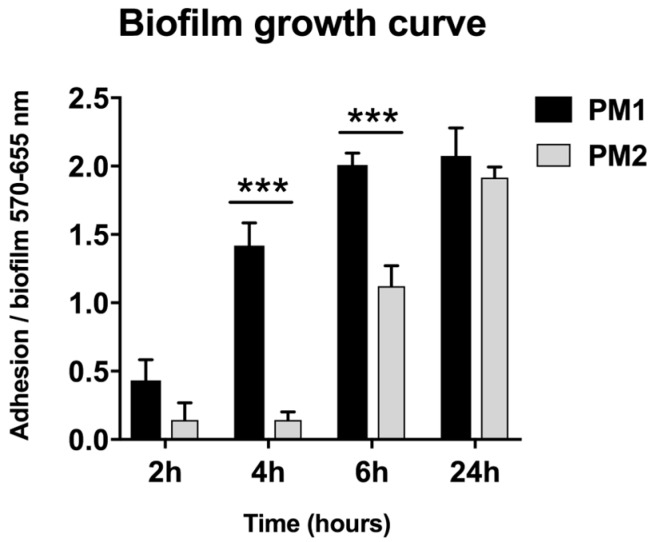
OD readings at 570–655 nm at various time points of biofilm growth of PM1 and PM2. Data are mean ± SD. Significant differences are indicated by *** *p* < 0.001 for comparison of PM1 vs. PM2.

The biofilm mode of growth confers on the associated organisms a measurable decrease in antimicrobial susceptibility; for this reason, it is essential to know the times of growth and biofilm development. The ability of the strains to form biofilms on abiotic surfaces was evaluated by the crystal violet test [21]. Here we show that both PM1 and PM2 strains were able to adhere and form biofilms on abiotic surfaces, but with different kinetics (Figure 3). The PM1 strain adhered and began to multiply and form biofilms already after 2 h of incubation, reaching the morphology of mature biofilms, a multilayer film that shows crystals of magnesium ammonium phosphate (termed struvite) and of hydroxylated calcium phosphate (termed apatite) after 6 h of incubation. The PM2 strain formed mature biofilms only after 24 h of incubation (Figure 3). Successful access of the bacterial cells and biofilm formation was confirmed by counting the viable sessile cells (PM1 = 2.2 × 10^8^ CFU/mL and PM2 = 1.5 × 10^8^ CFU/mL) and the number of planktonic cells (PM1 = 4.6 × 10^9^ CFU/mL and PM2 = 6.7 × 10^9^ CFU/mL).

**Figure 4:**
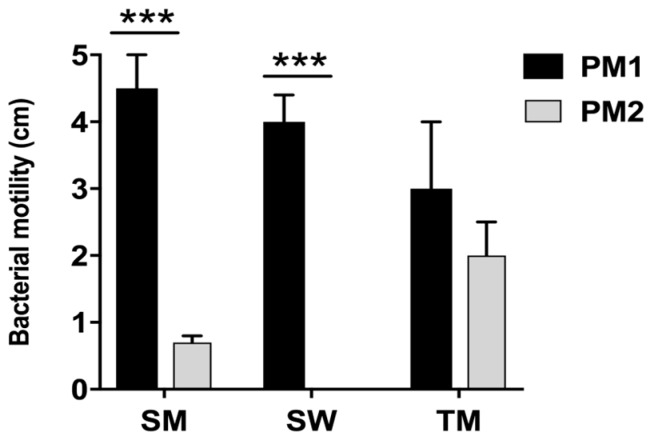
Evaluation of swimming (SM), swarming (SW) and twitching (TM) motility in PM1 and PM2. Data are mean ± SD. Significant differences are indicated by *** *p* < 0.001 for comparison of PM1 vs. PM2.

The PM1 and PM2 strains also had different movement skills. Motility is an important virulence factor since it allows the microorganisms, in the course of infection, to be disseminated from the site of colonization to other areas of the urinary tract. PM2 on 0.7% agar showed a tendency not to migrate even though swimming and twitching tests showed that it was mobile; conversely, PM1, already defined as swarmer for its ability to swarm on CLED agar medium, showed high motility (Figure 4).

**Figure 5:**
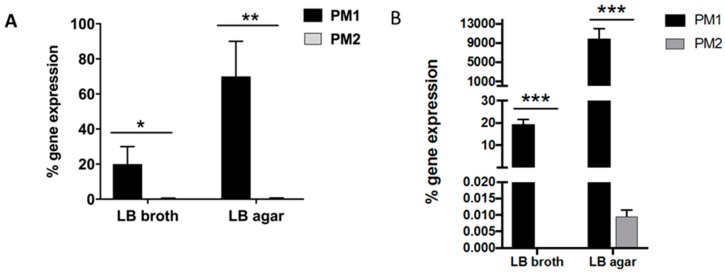
*wosA* (**A**); and *flhDC* (**B**) gene expression in PM1 and PM2 growth in LB broth or agar; data are expressed as a percentage of mRNA levels in each group normalized to 16S RNA. Data are mean ± SD. Significant differences are indicated by * *p* < 0.05, ** *p* < 0.01 and *** *p* < 0.001 for comparison of PM1 vs. PM2.

It is known that a “swarming” phenotype is associated with the increased expression of the *wosA* gene, which is able to upregulate the transcription of *flhDC*, which is essential for the regulation of a flagellar cascade, from four to 16 times [12]. We therefore evaluated the *wosA* and *flhDC* gene expression in the two strains (Figure 5). In PM2, the expression of both genes was not upregulated, while the PM1 strain showed higher levels of expression already in the liquid medium. The *wosA* and *flhDC* expression levels increased dramatically when the PM1 strain was grown on solid medium (Figure 5).

### 2.3. Evaluation of the Host Inflammatory Response

After the infection of T24 cells with PM1 and PM2 strains, levels of some immunoregulatory cytokines were examined, as the proinflammatory response is a key factor in inducing chemotaxis of immune cells and producing biomolecules at the site of infection.

Quantitative PCR was performed to evaluate the expression of IL-6, IL-8, IL-1β, TGF-β cytokines and of the antimicrobial peptide HBD-2 T24 cells infected with PM1 and PM2 relative to uninfected control cells.

**Figure 6:**
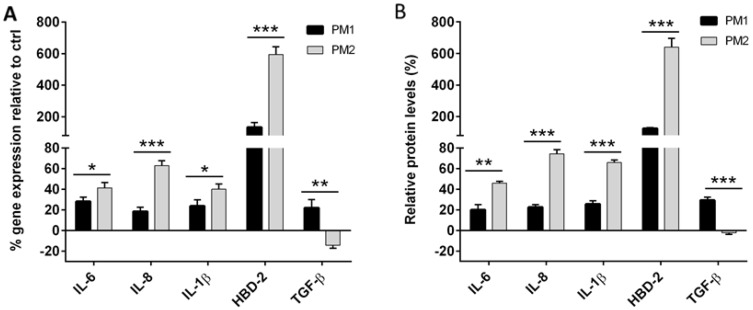
Relative gene expression (**A**) and protein concentration (**B**) in T24 cells infected with PM1 or PM2. Data are mean ± SD and are expressed as percentage of increment relative to unstimulated T24 cells (ctrl). Significant differences are indicated by * *p* < 0.05, ** *p* < 0.01, *** *p* < 0.001 for comparison of PM1 vs. PM2.

Our results indicate that PM1 induced low levels of proinflammatory cytokines IL-6, IL-8 and IL-1β, and of the antimicrobial peptide HBD-2, but was able to stimulate high concentrations of the anti-inflammatory cytokine TGF-β, whereas PM2 induced a strong inflammatory response by stimulating higher levels of IL-6, IL-8, IL-1β and HBD-2, but did not induce the expression of TGF-β (Figure 5 and Figure 6).

## 3. Discussion

*P. mirabilis* is, after *Escherichia coli*, the most frequent causative agent of catheter-associated urinary tract infections, especially in patients with urogenital anomalies [9].

This microorganism, a Gram-negative bacillus, is found in the human intestinal tract as part of the normal human intestinal flora, and is associated with complicated infections leading to urethritis, cystitis, acute pyelonephritis, chronic inflammation and bacteremia [8,16,17].

During the first 24 h after infection in the bladder, *P. mirabilis* performs its inflammatory action by colonization, due to fimbriae and the enzyme urease, an extracellular niche in which large clusters form. These clusters are non-uniform and, in addition to bacteria, contain urothelial cell debris, mineral deposits and neutrophils [22].

Bacterial adhesins recognize receptors on the surface of the host cells, and when the bacteria are firmly adhered, they undergo phenotypic changes and produce exopolysaccharides that trap and protect them [9]. In addition, urease breaks down the urea present in high concentrations in the urine into ammonia and bicarbonate, which causes an alkalinization of the urine and leads to the precipitation of polyvalent ions assembled with the microorganisms [19].

The bacteria multiply and form microcolonies that can mature into the so-called crystalline biofilms that protect them from antibiotics and the immune response [7].

The aim of this study was to assess the ability of the two phenotypically different, clinically isolated strains of *P. mirabilis*, PM1 and PM2, to produce biofilm and induce an innate immune response in monolayers of T24 cells.

First, our data indicate that the two strains have a different mobility. Specifically, while the PM1 strain was defined as a swarmer thanks to its remarkable ability to move, PM2 showed a tendency not to migrate on 0.5% agar.

It is known that an overswarming phenotype is associated with an increase in the expression of the *wosA* gene, which in turn is able to upregulate the transcription of *flhDC*, which is essential for the activation of the flagellar cascade, from 4 to 16 times [12].

PM2 did not express high levels of this gene, while it was highly expressed in PM1, and its expression increased in passing from a liquid to a solid medium. During growth in the liquid medium, PM1 presented as a single mobile cell of 1–2 μm in length, with a distinct phenotype seen by the presence of peritrichous flagella on the cell surface and the ability to move in the liquid medium (swimming motility). When transferred to a solid medium, these cells differentiated into hyperflagellate swarming forms, measuring from 20 to 80 μm in length. They migrated from the injection site quickly and in a coordinated manner through multi-cellular interactions and cell–cell signaling.

Conversely, an absence of movement of the PM2 strain observed in the swarming test was confirmed by the morphology of non-swarming cells.

However, despite the different degree of mobility and the difference in gene expression, both strains were capable of forming biofilm and this may be important for the establishment of infection in catheterized patients. In particular, the PM1 strain, probably due to its high motility, was able to colonize quickly and grow in biofilms after 6 h of incubation, whereas the PM2 strain formed a biofilm only after 24 h of incubation.

Biofilms are recognized as the ultimate cause of persistent and destructive infections and inflammatory processes, and this different organization in biofilm formation consequently involves a different efficiency in the host immune response, thus affecting the severity of infection.

The differences between the two strains are also related to a different modulation in T24 (human urinary bladder transitional cell carcinoma) cells of inflammation mediators such as pro-inflammatory cytokines IL-6, IL-8 and IL-1β and the anti-inflammatory cytokine TGF-β. We chose to use T24 cells as they have been shown to be similar to primary human bladder epithelial cells, first in that they express Toll-like receptor 4 and therefore they are sensitive to stimulation by lipopolysaccharide (LPS), and also because they have been used in several studies as responsive to infection with Gram-negative bacteria commonly associated with UTIs [21,22].

Inflammation is characterized by the interplay between proinflammatory and anti-inflammatory cytokines. Epithelial cells produce several immunoregulatory cytokines after bacterial insults contributing to host inflammatory and immune responses [23]. The orchestrated strategies evoked by epithelial cells are consequences of several factors, including the site of infection and the specific pathogen. Proinflammatory cytokines are involved in the upregulation of the host immune response by promoting systemic inflammation and acting to worsen the infection; on the other hand, the anti-inflammatory cytokines are a series of immunoregulatory molecules that modulate the intensity of the inflammatory process and contribute to its resolution.

In addition to cytokines, the antimicrobial peptide HBD-2 is also of great importance in the mechanisms involving the innate immune response. This peptide shows a very broad spectrum of microbicidal activity, acts on Gram-positive and Gram-negative bacteria, fungi and the envelopes of some viruses, and is involved in the innate immune response because its release is induced by proinflammatory cytokines and invasive microorganisms.

Our data indicate that PM1 induces low levels of proinflammatory cytokines IL-6, IL-8 and IL-1β, and the antimicrobial peptide HBD-2, but is able to stimulate, after only 6 h of infection, the expression of the anti-inflammatory cytokine TGF-β. These data suggest that PM1 potentially promotes the development and chronicity of the damage, which could result in cell death by apoptosis.

Conversely, PM2 is able to induce a strong inflammatory response by stimulating higher levels of IL-6, IL-8, IL-1β and HBD-2, but does not induce the expression of TGF-β; thus, by failing to exert an anti-inflammatory effect, the inflammatory response triggered by the pathogen is not deleted prematurely.

Overall, we found several differences in the expression of key virulence factors between the two studied *P. mirabilis* strains. PM1 shows an increased motility, overexpression of the *wosA* gene and a rapid growth in biofilm, all factors important for the establishment, growth and dissemination of uropathogens in the urinary tract. Moreover, probably due to the ability of rapid organization in biofilm, PM1 seems to be able to evade the recognition system of the epithelial cells.

It is well established that biofilms are inherently more resistant to antimicrobial treatments and they have been shown to tolerate much higher doses than planktonic bacteria [24]. In fact, many chronic infections in humans are thought to be related to biofilms. Typical biofilm-associated diseases are recurrent urinary tract infection, cystic fibrosis, periodontitis, endocarditis, and chronic wounds [25]. In addition, bacterial biofilm can also attenuate the immune response, thus evading clearance and persisting within the host. The PM1 strain has been shown to be able to form biofilm faster than PM2, and also is able to stimulate a lower inflammatory response. This makes us speculate that the infection of PM1 evolves towards chronicity.

In our past studies, we have also shown that *Pseudomonas fluorescens*, an opportunistic pathogen closely related to *Pseudomonas aeruginosa*, behaves in a different way according to the growth temperature, and especially when forming biofilms, induces a lower inflammatory response in lung epithelial cells [26].

In conclusion, both complicated and uncomplicated UTI is a significant health problem in the community. It typically leads to embarrassment, curtailment of daily, social activities and is a considerable economic burden on the individual as well as on the healthcare system.

The identification and understanding of the virulence factors of bacteria that cause these infections are necessary for their prevention and treatment, while a deeper understanding of the relations between the microorganism and host may provide the basis for new solutions to these clinical problems as regards both the diagnosis and therapy. This is very important also in the prevention of irreversible kidney damage, prolonged treatment, complications, as well as in the recurrence and chronicity of the infection.

## 4. Materials and Methods

### 4.1. Bacterial Strains

The *P. mirabilis* strains (PM1 and PM2) were isolated in 2009 from patients with infectious diseases of the renal system at the Bacteriology Division of the “Unità Operativa di Microbiologia e Virologia” of the Medical School of the Second University of Naples. These strains were first identified by the API20NE identification system (BioMèrieux, Craponne, France), and then confirmed by partial DNA sequencing of the 16S–23S rRNA gene using primers wl-5793 (5′-TGT ACA CAC CGC CCG TC-3′) and wl-5794 (5′-GGT ACT TAG ATG TTT CAG TTC-3′) [27] and finally by BLAST analysis.

All strains were grown in Luria-Bertani medium (LB) and on CLED agar plates at 37 °C in anaerobic conditions.

### 4.2. Growth Curve of a Planktonic Population

The overnight cultures of PM1 and PM2 were 40-fold diluted in LB medium and incubated at 37 °C with constant shaking. Every 60 min the optical density was measured at 600 nm (BioPhotometer Eppendorf, Milan, Italy) and the colony forming units (CFUs) were counted by spreading serial dilutions on LB agar plates and incubating at 37 °C overnight.

### 4.3. Biofilm Growth Curve

For the biofilm assay, overnight cultures of PM1 and PM2 were diluted to obtain a concentration of 107 CFUs/mL, and aliquots (200 μL) of the diluted bacterial suspension were placed into 96-well flat-bottomed sterile polystyrene microplates (Costar, Corning, Inc., New York, NY, USA) and incubated overnight at 37 °C in anaerobic conditions. The biofilm formed was quantified by a modification of the crystal violet assay [28]. Briefly, after 2, 4, 6 and 24 h the attached bacteria were washed twice with 200 μL of PBS and air-dried for 45 min. The wells were then stained with 200 μL of 1% aqueous crystal violet solution for 45 min. The plates were rinsed with 200 μL of sterile distilled water to remove excess dye and air-dried. The dye associated with attached biofilm was dissolved in a solution of 200 μL of ethanol, and the OD570/655 absorbance was measured on a microplate reader (580 Bio-rad, Laboratories, Segrate, Italy).

### 4.4. Swarming, Swimming and Twitching

Swimming. Tryptone swim plates (1% tryptone, 0.5% NaCl, 0.3% agar) were inoculated with PM1 and PM2 using a sterile needle and incubated at 30 and 37 °C for 24 h.

Swarming. Swarm plates were composed of 0.5% agar and 0.8% nutrient broth, supplemented with 0.5% glucose, and dried at room temperature overnight before use. Plates were inoculated with a sterile needle and incubated at 30 and 37 °C for 48 h.

Twitching. Bacteria were inoculated with a sterile needle through the twitch agar (1% TSB, 1% agar) to the bottom of the Petri dish. After incubation at 30 and 37 °C for 72 h, a hazy zone of growth at the interface between the agar and the polystyrene surface was observed. After carefully removing the agar layer, the unattached cells were eliminated by washing with distilled water and the attached bacteria stained with 1% crystal violet solution. Motility was assessed by calculating the diameter (mm) of the circular turbid zone formed by the bacterial cells migrating away from the point of inoculation. All tests were performed in quadruplicate and repeated on two different occasions.

### 4.5. Evaluation of the wosA Gene Expression Evaluation of the wosA and flhDC Gene Expression

To evaluate *wosA* and *flhDC* gene expression by PM1 and PM2, 5 μL of overnight cultures of planktonic and swarming bacteria were inoculated in 20 mL of LB broth or on LB-agar 0.7%, respectively, and incubated at 37 °C for 24 h. After incubation, aliquots of each sample (OD_600_ = 1.0) were used for total RNA extraction with High Pure RNA Isolation Kit (Roche; Milan, Italy). Two hundred nanograms of total cellular RNA were reverse-transcribed (Expand Reverse Transcriptase, Roche; Milan, Italy) into complementary DNA (cDNA) using random hexamer primers (Random hexamers, Roche; Milan, Italy) at 42 °C for 45 min, according to the manufacturer’s instructions.

PCR analysis of the *wosA* and *flhDC* gene expression was performed in a GeneAmp PCR system 9700 (Applied Biosystems, Foster City, CA, USA) and hot start GoTaq polymerase (Promega, Madison, WI, USA); 16S RNA was used as a control gene. The sequences of primers used were: *wosA* Fw: 5′-CTGCAATGCCAGCAACAACT-3′; *wosA* rev: 5′-AACCTTGTTGGCAGCCTTCT-3′; *flhDC* Fwd: 5′-CGCACATCAGCCTGCAAGT-3′; *flhDC* rev: 5′-GCAGGATTGGCGGAAAGTT-3′. PCR conditions were: initial denaturation at 95 °C for 5 min followed by 35 cycles: 95 °C for 30 s, 53 °C for 30 s and 72 °C for 1 min with a final extension at 72° for 10 min. The amplification products, respectively 477 and 90 bp, were analyzed on 1.6% agarose gel to control the amplicon lengths.

### 4.6. Cell Culture and Treatment

T24 cells (epithelioid carcinoma cells of human urinary bladder) were cultured in McCoy’s 5a Medium (Gibco) supplemented with 1% Penstrep, 1% glutamine and 10% fetal calf serum (Invitrogen, Waltham, MA, USA) at 37 °C in air and 5% CO_2_. Subsequently, cells were dispensed into six-well plates and left to grow until 80% of confluence.

Semiconfluent monolayers were then infected with exponentially-growing PM1 and PM2 at a multiplicity of infection (MOI) of 5 bacteria/cell. Infection was carried out for 6 (for gene expression analysis) and 24 h (for ELISA assay) at 37 °C in 5% CO_2_.

By preliminary test of gentamicin protection, both strains of *P. mirabilis* have shown to have poor invasive capacity, so it was therefore considered adhesiveness only.

In order to evaluate the expression of pro- and anti-inflammatory cytokines, the cells were then washed three times with sterile PBS, and total RNA was extracted using High Pure RNA Isolation Kit (Roche Diagnostics, Monza, Italy).

### 4.7. Real-Time PCR

Two hundred nanograms of total cellular RNA were reverse-transcribed (Expand Reverse Transcriptase, Roche; Milan, Italy) into complementary DNA (cDNA) using random hexamer primers (Random hexamers, Roche; Milan, Italy) at 42 °C for 45 min, according to the manufacturer’s instructions.

Real time PCR for IL-6, IL-8, TNF-α, IL-1α, IL-1β, TGF-β and HBD-2 was carried out with the LC Fast Start DNA Master SYBR Green kit using 2 μL of cDNA, corresponding to 10 ng of total RNA in a 20 mL final volume, 3 mM MgCl_2_ and 0.5 mM sense and antisense primers (Table 1).

After amplification, melting curve analysis was performed by heating to 95 °C for 15 s with a temperature transition rate of 20 °C/s, cooling to 60 °C for 15 s with a temperature transition rate of 20 °C/s, and then heating the sample at 0.1 °C/s to 95 °C. The results were analyzed using LightCycler software (Roche Diagnostics GmbH, Monza, Italy). The standard curve of each primer pair was established with serial dilutions of cDNA. All PCR reactions were run in triplicate.

The specificity of the amplification products was verified electrophoresis on a 2% agarose gel and visualization by ethidium bromide staining.

### 4.8. ELISA Assay

T24 cell monolayer were infected with PM1 and PM2 for 24 h at 37 °C, as described above. At the end of the experiment, supernatants were harvested and the presence of cytokines IL-6, IL-8, IL-1β, and antimicrobial peptide β-defensin 2 (HBD-2) was analyzed by enzyme-linked immunosorbent assay (ELISA; ThermoFischer Scientific Inc., Waltham, MA, USA; Phoenix Pharmaceuticals Inc., Burlingame, CA, USA).

### 4.9. Statistical Analysis

All statistical analyses were performed using Student’s *t*-test. *p*-value < 0.05 was considered statistically significant. Significant differences between PM1 and PM2 were indicated in figures by * *p* < 0.05, ** *p* < 0.01, *** *p* < 0.001. The data are means ± standard deviation (SD) of three independent experiments.

## 5. Conclusions

In this work we have identified and characterized two clinical isolate strains that have expressed different phenotypes and characteristics. The results may be interesting from the point of view of clinical and diagnostic practice, and useful for a correct therapeutic approach; however, investigations to clarify the mechanisms underlying this diversity are currently being targeted.

## Figures and Tables

**Table 1:** Primers sequences.

Gene	Primers Sequence	Conditions	Product Size (bp)
*IL-6*	5′-ATGAACTCCTTCTCCACAAGCGC-3′ 5′-GAAGAGCCCTCAGGCTGGACTG-3′	5″ at 95 °C, 13″ at 56 °C, 25″at 72 °C for 40 cycles	628
*IL-8*	5′-ATGACTTCCAAGCTGGCCGTG-3′ 5′-TGAATTCTCAGCCCTCTTCAAAAACTTCTC-3′	5″ at 94 °C, 6″ at 55 °C, 12″ at 72 °C for 40 cycles	297
*IL-1β*	5′-GCATCCAGCTACGAATCTCC-3′ 5′-CCACATTCAGCACAGGACTC-3′	5″ at 95 °C, 14″ at 58 °C, 28″ at 72 °C for 40 cycles	708
*TGF-β*	5′-CCGACTACTACGCCAAGGAGGTCAC-3′ 5′-AGGCCGGTTCATGCCATGAATGGTG-3′	5″ at 94 °C, 9″ at 60 °C, 18″ at 72 °C for 40 cycles	439
*hBD-2*	5′-GGATCCATGGGTATAGGCGATCCTGTTA-3′ 5′-AAGCTTCTCTGATGAGGGAGCCCTTTCT-3′	5″ at 94 °C, 6″ at 63 °C, 10″ at 72 °C for 50 cycles	198
